# The role of the socio-economic environment on medical outcomes after ST-segment elevation myocardial infarction

**DOI:** 10.1186/s12889-019-6966-z

**Published:** 2019-05-23

**Authors:** Christian Roth, Rudolf Berger, Michael Kuhn

**Affiliations:** 1 Department of Internal Medicine II, Cardiology, Medical University of Vienna, Waehringer Guertel 18-20, 1090 Vienna, Austria; 2 Department of Internal Medicine I, Cardiology and Nephrology, Hospital of St. John of God, Johannes von Gott-Platz 1, 7000 Eisenstadt, Austria; 3 Wittgenstein Centre (WU, VID/ÖAW, IIASA), Institute for Applied Systems Analysis and Vienna Institute of Demography, Welthandelsplatz 2/Level 2, 1020 Vienna, Austria

**Keywords:** ST-segment elevation myocardial infarction, Socio-economic environment, Conventional risk factors

## Abstract

**Background:**

According to the World Health Organization, coronary artery disease (CAD), including ST-segment elevation myocardial infarction (STEMI), is the most common cause of death worldwide as well as in Europe and Austria. There is valid data on the impact of conventional risk factors on the medical outcomes for STEMI patients. However, only few studies examine the role of the socio-economic environment for medical outcomes. The main task of this study is to investigate if the socio-economic environment of patients who underwent percutaneous coronary intervention (PCI) after STEMI has an impact on the distribution of risk factors and medical outcomes.

**Methods:**

The study focuses on the population of the City of Vienna, Austria, and includes 870 STEMI patients, who underwent PCI at the General Hospital of Vienna (AKH Wien) between 2008 and 2012. The following data were collected: conventional risk factors (hypertension, hyperlipidemia, diabetes, overweight, smoking, family history and vascular disease) and socio-economic indicators of the patient’s residential district (number of residents, income pre-tax, residents per general practitioner, residents per internal specialist, compulsory education only, academic degree and rate of unemployment). Cox regressions were performed to evaluate the impact of socio-economic environment and conventional risk factors on survival.

**Results:**

Most of the conventional risk factors show a significant difference between deceased and surviving patients. The study revealed significant differences across districts in relation to the socio-economic background of STEMI patients. Surprisingly, medical outcomes, as measured by the survival of patients, are significantly related to a patient’s district of residence (*p*-Value = 0.028) but not in a systematic way as far as the socio-economic environment of these districts is concerned.

**Conclusions:**

The study provides intuitive evidence for a hitherto understudied Central European context on the link between socio-economic environment and conventional risk factors at population level and the link between conventional risk factors and survival both at the population at the individual level. While this is in line with previous evidence and suggestive of the incorporation of measures of socio-economic status (SES) into policy & guidelines toward the management of CAD, more data on the SES – STEMI nexus are needed at individual level.

## Background

According to the World Health Organization (WHO), coronary artery disease (CAD) is the most common cause of death worldwide. In 2015, 8.76 million people died because of CAD, amounting to 15.5% of all deaths [1]. According to the “Heart disease and stroke statistics 2016” by the American Heart Association (AHA), the total prevalence of CAD in the United States is 6.2% in adults aged 20 years and over. CAD prevalence is 7.6% in men and 5.0% in women [2]. In Europe, every sixth man and every seventh woman is dying from myocardial infarction [3]. Diseases of the cardiovascular system are the most frequent cause of death in Austria as well. A recent report by Statistik Austria shows that 37.8% of all male decedents and 47.4% of all female decedents died because of CAD [4].

Several risk factors are responsible for the appearance of CAD and the potential risk of ST-segment elevation myocardial infarction (STEMI). Conventional risk factors, including hypertension, hyperlipidemia, diabetes mellitus, overweight, smoking, family history, cerebrovascular disease (CVD), peripheral vascular disease (PVD) and age, influence the occurrence and progression of CAD. Therefore, they have a clear impact on the prognosis and medical outcome for these patients [5–14].

Aside from the conventional risk factors, there is a notable influence of a person’s socio-economic status (SES). In general, SES is the extent of a person’s access to collectively desired resources, such as material goods, money, power, friendship networks, healthcare, leisure time or educational opportunities, and is a fundamental aspect in social and health sciences [15, 16]. Investigations of SES and statistics based on indicators of SES are necessary to quantify the level of social stratification or inequality in or between societies. These analyses are important to capture and understand changes of social structures and the intergenerational change of social status over time. Finally, SES matters because it is related to health and life outcomes and is therefore an important determinant of the performance of health care systems [15, 16]. Studies have shown that a low SES in patients with CAD is associated with a higher rate of conventional risk factors, unfavorable health in general and more cardiovascular events such as STEMI [17–21]. It is also associated with a higher morbidity, mortality and more complications after heart surgery like coronary artery bypass grafting [22–25]. A low SES has also been linked to disproportional use of health care in regard to other diseases [26].

Depending on the availability of data, SES is measured either at the individual level or in a more summarily way at the level of the individual’s residential environment or neighbourhood [27–29]. Studies based on such “neighbourhood” indicators have found a significant impact of the socio-economic environment on individual health behaviour and outcomes for several regions and cities [17, 20, 30]. This includes health outcomes in respect to CAD [18, 21, 23].

The place of residence gives information about the socio-economic environment of the patient. It also shows the distance from the patient’s home to a hospital, as it is important to perform PCI in STEMI patients as quickly as possible. Patients who live closer to a hospital have an advantage compared to those who live farther away [31, 32]. Furthermore, the place of residence shows if a patient lives in a favorable area of a city, reflecting the socio-economic surroundings.

A relationship between SES and health behaviours, such as smoking and drinking, and outcomes, such as morbidity and mortality for chronic disease and, notably, coronary heart disease, has been observed for a long time [30, 33]. Strikingly, the vast majority of these studies are set against the background of Anglo-Saxon countries (US, UK and Australia) with few additional studies from Scandinavia. In contrast, insights on the impact of SES on the outcomes after STEMI [21] are sparse, especially for continental European countries with their distinct social and welfare state environment.

This study seeks to provide some insights by studying the relationship between the socio-economic environment at residential district level in the City of Vienna, Austria, and (i) the prevalence of conventional risk factors at district level, as well as (ii) health outcomes for a population of patients undergoing PCI after STEMI at the Vienna General Hospital as a single high volume center. The aims are to identify correlations between SES environment and the prevalence of risk factors and to establish whether residential district has an impact on the medical outcomes for STEMI patients. An understanding of the role of socio-economic inequalities for medical outcomes is important to better target health care resources and to provide optimal medical treatment for patients undergoing PCI after STEMI.

## Methods

### Study population

All patients presented with STEMI on Saturday or Sunday at the AKH Wien within the time span 2008 until 2012 were included in the study. Restricting the inclusion to weekend days was a deliberate decision, as the AKH Wien is the only hospital in charge of the treatment of STEMIs during weekends. Hence it is guaranteed that the study population represents a balanced mix of patients from all over Vienna.

Each coronary angiogram was executed by experienced interventionalists. The coronary angioplasty and the medical treatment were achieved according to the ESC guidelines [3].

After clarifying the survival status, all patients in the database were anonymized using a unique patient identification (ID). This study is approved by the ethics committee of the Medical University of Vienna (MUW) (EK Nr. 972/2011) and is also in line with the Declaration of Helsinki.

### Endpoint

The primary endpoint of the study was the survival of patients. To determine date and cause of death of each patient the database was synchronized with the Austrian Death Registry database (Statistik Austria) at the end of the follow-up period. The due day of the data requested was the 31st of December 2014.

### Data collection

Suitable patients were identified via the corresponding database of the catheter laboratory (Cardio-Report). The angiographic characteristics were collected from this database. Baseline characteristics and conventional risk factors were collected from the hospital information system (Krankenhausinformationssystem – KIS). All data was aggregated in an already existing comprehensive database, which has been built in close cooperation with the Center for Medical Statistics, Informatics, and Intelligent Systems (CeMSIIS) of the MUW using the Research Documentation and Analysis (RDA) IT-system.

### Socio-economic environment

Measures of the socio-economic environment in this study are based on the postal code of each patient. A first set of socio-economic indicators includes: place of residence, pre-tax income, educational background (compulsory education only, academic degree) and rate of unemployment. A second set relates to the primary health care system, measuring the per capita number of general practitioners and internal specialists of the area the patient lives in. These data were collected for each of Vienna’s districts from Statistik Austria, Ärztekammer Wien and “Stadt Wien, Magistratsabteilung 23 – Wirtschaft, Arbeit und Statistik” [34–43]. To avoid statistical bias due to the development of the districts over time, all data was collected for each individual year from 2008 through 2012.

### Statistical analysis

The statistical analysis was performed by using SPSS (Version 24.0). Categorical variables are shown as counts or percentages, as appropriate. Categorical data is compared by crosstabs using Pearson’s chi-square test. Continuous variables are shown as median with interquartile range (IQR) or mean ± SD, as appropriate, and are compared using ANOVA. To detect a possible relation between socio-economic environment and place of residence the correlation was calculated. To evaluate the impact of variables on survival, Cox regression models were calculated. Kaplan-Meier analysis was used to estimate the cumulative probability of reaching an endpoint (survival) and the log-rank test was used to assess survival differences. All *p*-values under 0.05 are considered statistically significant. To analyze survival over a longer time span and to employ a bigger population sample, the population examined in the survival analysis was extended to also include STEMI patients from 2000 to 2007. Thus, 1605 patients with 356 endpoints were included in the survival analysis covering the overall time span 2000–2012.

## Results

### Baseline characteristics

A total of 870 patients with STEMI -- 613 male and 257 female patients with a mean age of 62 (±13.83) years -- underwent PCI in a high volume center (AKH Wien) between 2008 and 2012. The baseline characteristics of the patient population, including epidemiological data and conventional risk factors, are given in Table 1.

**Table 1:** Patient characteristics

	total *n* = 870
Baseline characteristics	
Age, years; mean (±SD)	61.73 (±13.83)
Age > 62; n (%)	416 (47.8)
Gender	
- Female; n (%)	257 (29.5)
- Male; n (%)	613 (70.5)
STEMI per year	
- 2008; n (%)	199 (22.9)
- 2009; n (%)	113 (13.0)
- 2010; n (%)	210 (24.1)
- 2011; n (%)	202 (23.2)
- 2012; n (%)	146 (16.8)
Height, cm; mean (±SD)	172.58 (±0.091)
Weight, kg; mean (±SD)	83.18 (±16.821)
Body-Mass-Index, kg/m^2^; mean (±SD)	27.7 (±5.037)
Overweight (BMI > 25); n (%)	404 (76.8)
Heart rate, bpm; mean (±SD)	74.35 (±14.488)
Hypertension; n (%)	504 (57.9)
Systolic blood pressure, mmHg; mean (±SD)	128.87 (±20.002)
Diastolic blood pressure, mmHg; mean (±SD)	77.32 (±13.093)
Diabetes mellitus	
- NIDDM; n (%)	107 (12.3)
- IDDM; n (%)	46 (5.3)
Hyperlipidemia; n (%)	474 (54.5)
Smoker	
- current; n (%)	319 (36.7)
- previous; n (%)	88 (10.1)
Family history; n (%)	135 (15.5)
Central vascular disease; n (%)	57 (6.6)
Peripheral vascular disease; n (%)	45 (5.2)
Prior myocardial infarction; n (%)	92 (10.6)
Prior PCI; n (%)	77 (8.9)
Prior coronary artery bypass graft; n (%)	17 (2.0)
NYHA class	
- Class I; n (%)	388 (44.6)
- Class II; n (%)	29 (3.3)
- Class III; n (%)	45 (5.2)
- Class IV; n (%)	284 (32.6)
CCS class	
- Class I; n (%)	20 (2.3)
- Class II; n (%)	28 (3.2)
- Class III; n (%)	54 (6.2)
- Class IV; n (%)	676 (77,7)
Angiographic characteristics	
Vessel disease	
- 1VD; n (%)	459 (52.8)
- 2VD; n (%)	238 (27.4)
- 3VD; n (%)	140 (16.1)
- LM only; n (%)	33 (3.8)
Number of procedures	
- one; n (%)	618 (71.0)
- two; n (%)	173 (19.9)
- three; n (%)	60 (6.9)
- > three; n (%)	19 (2.2)
Patients treated with	
- drug-eluting stent; n (%)	849 (97.6)
- bare-metal stent; n (%)	21 (2.4)
Re-catheterizations	
- < 30 days; n (%)	167 (19.2)
- 30 days – 6 months; n (%)	32 (3.7)
- 6 months – 1 year; n (%)	30 (3.4)
- > 1 year; n (%)	23 (2.6)
Number of stents	
- one; n (%)	593 (68.2)
- two; n (%)	162 (18.6)
- three; n (%)	67 (7.7)
- > three; n (%)	48 (5.5)
Revascularization	
- total; n (%)	676 (77.7)
- partly; n (%)	194 (22.3)
Cause of death	
Total death; n (%)	167 (19.2)
Cardiovascular, acute coronary syndrome; n (%)	59 (6.8)
Cardiovascular, coronary artery disease; n (%)	45 (5.2)
Cardiovascular, vitium; n (%)	9 (1)
Cardiovascular, cardiomyopathy; n (%)	54 (6.2)

Height and weight were collected from 526 patients. The mean body-mass-index of the population was 27.7 (±5.037) kg/ m^2^ and more than three-fourths (76.8%) of the patients showed overweight with a BMI bigger than 25 kg/m^2^.

More than half of all patients (*n* = 870) showed up with hypertension (57.9%) and hyperlipidemia (54.5%). Table 1 shows that 17.6% of the patients suffered from diabetes mellitus and 46.8% were current or previous smokers. A positive family history of cardiovascular events was shown in 15.5% of all patients. Furthermore, 10.6% of the patients had suffered from a myocardial infarction before. The main symptom of the patients was chest pain, with 83.9% presenting with CCS class III or IV.

### Angiographic characteristics

The angiographic characteristics of the whole patient population are listed in Table 1, with values given in total and percentage. More than half of the population (52.8%) had a one-vessel disease in the coronary angiography and could be treated with a single procedure (71.0%). Almost all patients (97.6%) were treated with drug-eluting stents. About two thirds of the patients received a total revascularization (77.7%).

### Cause of death

By the end of the observation period, 167 patients (19.2%) had died. The due day was the 31st of December 2014. All deaths during the observational time were due to cardiovascular circumstances. Acute coronary syndrome (6.8%), cardiomyopathy (6.2%) and progression of coronary artery disease (5.2%) were the most common causes of death. All causes of death in total and percentages can be seen in Table 1.

### Incidence and mortality

Table 2 shows STEMI incidence and mortality (amongst STEMI patients) per district and age stratification. While the incidence rate relates to the residents per district, the mortality rate relates only to the STEMI’s per district.

**Table 2:** Incidence and mortality, conventional risk factors and socio-economic aspects by district

		Incidence and mortality				Conventional risk factors											Socio-economic aspects					
	Residents (n)	STEMI (n)	Incidence (%)	Death (n)	Mortality (%)	Hypertension (%)	Hyperlipidemia (%)	NIDDM (%)	IDDM (%)	Overweight (BMI > 25) (%)	Current smoking (%)	Previous smoking (%)	Family history (%)	CVD (%)	PVD (%)	Age > 62 (%)	Income pre-tax (€)	Residents per general practitioner (n)	Residents per internal specialist (n)	Compulsory education only (%)	Academic degree (%)	Unemployment rate (%)
Total^#^	1,692,400	870	0.051	167	19.20	57.9	54.5	12.3	5.3	76.8	36.7	10.1	15.5	6.6	5.2	47.8	30,200	1395	6033	22.8	21.4	4.5
District 1^*^	16,455	13	0.079	2	15.38	76.9	38.5	15.4	23.1	88.9	23.1	7.7	15.4	23.1	15.4	84.6	51,405	258	384	11.5	44.3	2.0
District 2	94,832	42	0.044	9	21.43	50.0	40.5	11.9	2.4	70.8	33.3	0	11.9	0	2.4	54.8	27,393	1257	5611	25.6	23.8	4.9
District 3	83,746	34	0.041	4	11.76	61.8	52.9	11.8	0	77.8	35.3	2.9	29.4	5.9	11.8	61.8	32,797	1294	2425	20.0	29.5	4.6
District 4	30,419	20	0.066	2	10.00	50.0	70.0	10.0	15.0	68.8	15.0	40.0	20.0	10.0	15.0	30.0	36,232	805	1667	14.5	38.4	3.3
District 5	52,501	25	0.048	6	24.00	48.0	52.0	20.0	4.0	80.0	52.0	4.0	24.0	8.0	4.0	40.0	26,809	1303	5644	25.2	24.9	5.0
District 6	29,353	16	0.055	4	25.00	56.3	43.8	0	12.5	71.4	6.3	12.5	0	12.5	6.3	75.0	32,615	663	1493	15.1	35.2	4.5
District 7	30,065	16	0.053	3	18.75	31.3	43.8	6.3	0	60.0	50.0	0	25.0	0	0	31.3	33,627	547	1608	13.5	39.4	3.3
District 8^*^	23,987	14	0.058	2	14.29	85.7	78.6	14.3	7.1	72.7	50.0	14.3	28.6	0	0	35.7	35,047	516	663	11.7	43.1	2.9
District 9^*^	39,305	28	0.071	3	10.71	78**.**6	46.4	10.7	3.6	58.8	39.3	7.1	28.6	17.9	0	39.3	33,391	663	517	14.2	39.7	3.1
District 10^**^	174,919	97	0.055	23	23.71	55.7	57.7	13.4	6.2	81.0	35.1	14.4	13.4	8.2	4.1	52.6	25,743	1695	7956	31.2	11.0	5.4
District 11^**^	88,675	36	0.041	8	22.22	66.7	52.8	13.9	13.9	100.0	27.8	8.3	8.3	2.8	5.6	61.1	26,632	2045	13,377	27.4	9.8	6.3
District 12	87,341	32	0.037	9	28.13	46.9	46.9	12.5	6.3	82.4	31.3	6.3	12.5	0	3.1	28.1	26,683	1347	5618	28.2	16.8	5.6
District 13^*^	50,978	18	0.035	4	22.22	55.6	44.4	5.6	11.1	57.1	44.4	0	11.1	5.6	11.1	50.0	41,961	649	1436	11.8	36.1	2.7
District 14	84,278	41	0.049	11	26.83	46.3	48.8	22.0	2.4	76.5	31.7	4.9	17.1	2.4	7.3	51.2	31,917	2078	3992	20.8	22.4	4.4
District 15^**^	70,915	26	0.037	2	7.69	61.5	57.7	11.5	0	92.3	42.3	11.5	7.7	3.8	3.8	38.5	23,239	1436	7110	31.0	17.7	6.0
District 16	94,858	49	0.052	6	12.24	65.3	55.1	10.2	4.1	69.0	38.8	10.2	24.5	8.2	6.1	36.7	26,372	1293	5306	28.8	18.5	5.3
District 17	52,364	34	0.065	5	14.71	61.8	55.9	14.7	0	80.8	50.0	11.8	20.6	0	0	26.5	28,736	1201	2903	24.7	24.2	4.8
District 18	47,595	23	0.048	6	26.09	69.6	56.5	21.7	4.3	81.8	26.1	8.7	17.4	13.0	4.3	43.5	36,110	679	1245	15.2	38.2	3.5
District 19	68,003	41	0.060	11	26.83	51.2	56.1	4.9	2.4	77.4	31.7	14.6	12.2	7.3	2.4	56.1	38,785	793	1144	14.7	33.2	2.9
District 20^**^	82,304	61	0.074	8	13.11	63.9	57.4	21.3	4.9	77.5	27.9	13.1	11.5	8.2	3.3	54.1	24,654	1631	7506	31.2	15.9	5.3
District 21	140,916	77	0.055	14	18.18	58.4	62.3	13.0	5.2	80.4	46.8	9.1	14.3	6.5	6.5	46.8	29,046	1755	11,747	22.9	12.8	4.4
District 22^**^	156,091	80	0.051	12	15.00	55.0	57.5	5.0	2.5	71.2	46.3	12.5	17.5	6.3	5.0	37.5	31,757	1869	8575	18.3	14.4	3.3
District 23	92,500	47	0.051	13	27.66	55.3	53.2	8.5	10.6	72.4	34.0	10.6	2.1	8.5	8.5	66.0	34,331	1203	6564	17.1	18.9	3.5

^*^ top group of districts in terms of socio-economic environment; ^**^ bottom group of districts in terms of socio-economic environment,

^#^ entries for socio-economic aspects are given as resident-weighted averages

Over the five year period covered, the 1st district (0.079% from 16,455, *n* = 13), the 9th district (0.071% from 39,305, *n* = 28) and the 20th district (0.074% from 82,304, *n* = 61) exhibit the highest incidence rates. The highest STEMI mortality rates are found in the 12th district (28.13% from 32, *n* = 9), 14th district (26.83% from 41, *n* = 11), 19th district (26.83% from 41, n = 11) and 23rd district (27.66% from 47, *n* = 13). The lowest STEMI mortality rates are found in the 4th district (10.00% from 20, *n* = 2), the 9th district (10.71% from 28, *n* = 3) and the 15th district (7.69% from 26, n = 2). Further details are presented in Table 2.

### Conventional risk factors

The distribution of the conventional risk factors per district is also shown in Table 2. The following conventional risk factors were collected: hypertension, hyperlipidemia, NIDDM, IDDM, overweight, current and previous smoking, family history, CVD, PVD and age over 62 years. The highest percentage of patients with hypertension can be found in the 1st district (76.9%) and in the 18th district (69.6%). The district with the highest rate of patients with hyperlipidemia is the 8th district (78.6%). The greatest share of patients with NIDDM and IDDM can be found in the 14th district (22% NIDDM) and in the 6th district (12.5% IDDM), respectively. Surprisingly, all patients of the 11th district were overweight (BMI > 25). The lowest percentage of overweight patients can be found in the 13th district (57.1%). With 52% of all patients being current smokers, the 5th district exhibits the highest prevalence of smoking (among STEMI patients). The 3rd, 8th and 9th districts show the highest shares (3rd district: 29.4%; 8th and 9th districts: 28.6%) of patients with a positive family history. Both, CVD and PVD are most prominent amongst patients in the 1st district (23.1% CVD, 15.4% PVD). The 1st district is also the district with the highest share of patients above 62 years of age (84.6%).

### Socio-economic environment

The following socio-economic indicators were collected at district level: number of residents, pre-tax income, residents per general practitioner, residents per internal specialist, share of population with compulsory education only, share of population with academic degree, and rate of unemployment. The data are summarized in Table 2. All values are shown in means per district over the course of 5 years, 2008–2012. When ranking districts according to their socio-economic environment, as measured by the indicators, ranks increase in terms of pre-tax income and the population share with academic degree, whereas they decrease in terms of the other indicators (bar the number of residents, which is neutral). In the following, we classify districts depending on how many times they are included among the top-5, respectively bottom-5, in terms of the socio-economic environment according to the six indicators. The correlation between the socio-economic environment and the district was significant for all indicators, with the exception of pre-tax income and compulsory education only.

The following can be glanced from the table. The 1st district plays a special part, as it has the lowest number of residents but leads on all scores: It has by a large margin the highest income (€ 51,405); exhibits the lowest number of residents per general practitioner (*n* = 258) and per internal specialist (*n* = 384); it features the lowest share of those with compulsory education only (11.5%) and the highest share of those with academic degree (44.3%) as well as the lowest unemployment rate (2.0%). Other districts with high ranks in terms of socio-economic environment are the 8th and 9th district, both of which are among the top-5 districts according to 5 out of 6 indicators, as well as the 13th district (4 out of 6 in the top-5). At the opposite end, the 10th and 11th district are included among the bottom-5 districts for all indicators, the 20th district is included among the bottom-5 for 5 out of 6 indicators, and the 15th and 22nd district for 4 out of 6 indicators each. The lowest income (€ 23,239) is found in the 15th district, the highest numbers of residents per general practitioner (*n* = 2078) and per internal specialist (*n* = 13,377) are found in the 14th and 11th district, respectively. The highest share of those with compulsory education (31.2%) and the lowest share of those with an academic degree (9.8%) are found in the 10th and 11th district, respectively. The highest unemployment rate (6.3%) shows for the 11th district. In summary, we can say that districts 1, 8, 9, 13 (marked with a single asterisk in Table 2) form a top group in terms of socio-economic environment, whereas districts 10, 11, 15, 20 and 22 (marked with a double asterisk in Table 2) form a bottom group.

### Socio-economic environment and conventional risk factors

Columns 2 and 3 of Table 3 report the distribution of conventional risk factors among the whole patient population and among those who died, respectively. As listed in the table (*p*-Value), the distribution of the conventional risk factors shows a significant difference between dead and surviving patients. Only hypertension and overweight showed no significant difference.

**Table 3:** Socio-economic aspects and conventional risk factors

	All patients (%)	Dead (%)	Income pre-tax (€)	Residents per general practitioner (n)	Residents per internal specialist (n)	Compulsory education only (%)	Academic degree (%)	Unemployment rate (%)
Average for all patients			30,318	1382	5942	22.7	21.6	4.4
Hypertension								
- no	42.1	48.5	30,246	1381	6023	22.8	21.3	4.4
- yes	57.9	51.5	30,370	1382	5883	22.7	21.9	4.4
*p*-Value		0.061						
Hyperlipidemia								
- no	45.5	63.5	30,443	1368	5825	22.7	22.1	4.4
- yes	54.5	36.5	30,213	1394	6040	22.7	21.2	4.4
- *p*-Value		< 0.001						
Diabetes mellitus								
- no	82.4	73.1	30,342	1383	5908	22.6	21.7	4.4
- NIDDM	12.3	16.7	29,529	1413	6002	23.9	21.0	4.5
- IDDM	5.3	10.2	31,775	1298	6326	22.0	22.3	4.4
- *p*-Value		< 0.001						
Overweight (BMI > 25)								
- no	23.2	31.3	30,920	1287	5348	21.5	23.1	4.2
- yes	76.8	68.7	30,261	1397	6172	22.9	21.1	4.5
- *p*-Value		0.114						
Smoker								
- no	53.2	77.2	30,423	1385	5817	22.7	21.7	4.4
- previous	10.1	5.4	30,384	1356	5925	22.6	21.3	4.4
- current	36.7	17.4	30,148	1385	6127	22.8	21.2	4.4
- *p*-Value		< 0.001						
Family history								
- no	84.5	94.0	30,283	1392	6056	22.8	21.3	4.4
- yes	15.5	6.0	30,508	1330	5321	22.4	23.4	4.4
- *p*-Value		< 0.001						
CVD								
- no	93.4	85.0	30,225	1386	5979	22.8	21.5	4.4
- yes	6.6	15.0	31,647	1319	5413	21.5	23.5	4.2
- *p*-Value		< 0.001						
PVD								
- no	94.8	91.0	30,240	1380	5939	22.8	21.6	4.4
- yes	5.2	9.0	31,747	1413	5991	21.7	22.2	4.3
- *p*-Value		0.019						
Age > 62								
- no	52.2	15.0	30,016	1359	5891	22.9	21.7	4.4
- yes	47.8	85.0	30.647	1.407	5.998	22.6	21.6	4.4
- *p*-Value		< 0.001						

Columns 4–9 of Table 3 report the mean values of the various SES indicators within the patient groups who exhibit (or not) certain conventional risk factors. Given that the indicators are measured at the district level, the mean values then reflect a weighted average of the SES indicators across districts, with the share of patients from district *i* within risk group *j* acting as weights applied to the respective value of the SES indicator for district *i*. As a benchmark for comparison we also present for each of the indicators the average across all regions.

As one would expect perhaps, the risk factors hyperlipidemia, overweight and current smoking are associated with a disadvantageous socio-economic profile: Patients exhibiting these risk factors tend to be drawn on average from districts with a lower than average income, above average number of residents per general practitioner and internal specialist, above average share of compulsory education, below average share of academic degrees and above average unemployment rate. Conversely, hypertension and CVD are associated with an advantageous socio-economic profile in all categories. In particular patients with CVD are associated with the highest rate of academic degrees (23.5%) and the lowest rate of compulsory education only (21.5%). Patients drawn from the age group above 62 tend to be drawn from districts with above average socio-economic environment. While for this group the number of general practitioners per residents is below average, this may be due to the fact that these patients come from districts which exhibit an above average number of internal specialists, the latter being more likely to operate in affluent districts.

While patients without diabetes mellitus tend to be drawn from districts with an above average socio-economic environment (the exception being once more the number of residents per general practitioner), an interesting observation pertains to patients presenting with diabetes mellitus: Very prominently, patients with NIDDM are associated with a strongly disadvantageous SES profile: this group is associated with the lowest income (€ 29,529), the highest rate of compulsory education only (23.9%), the lowest rate of academic degrees (21%) and the highest rate of unemployment (4.5%). NIDDM patients also tend to be drawn from districts with a high proportion of residents per general practitioner (1413 on average) and per internal specialist (6002 on average).

Conversely, patients with IDDM tend to be drawn from high income districts (€ 31,775 on average) and from districts with a low proportion of residents per general practitioner (1298 on average). They are also associated with a low rate of compulsory education only (22%) and a high rate of academic degrees (22.3%). These patients also tend to be drawn from districts with the highest incomes (€ 31,647).

### Survival analysis

To analyze the causal impact on patient survival of conventional risk factors measured at the individual patient level and the socio-economic environment a COX- regression was performed. The socio-economic environment was measured by the various SES indicators for the patient’s residential district as well as by a district dummy. While most of the conventional risk factors showed a significant influence on survival, the analysis revealed no influence of the SES indicators. The hazard ratios (HR), 95% intervals and *p*-values associated with each socio- economic aspect are presented in Table 4.

**Table 4:** COX-regression

	HR	low95	up95	*p*-Value
Socio-economic aspect				
Income pre-tax (€)	0.911	0.656	1.266	0.421
Residents per general practitioner (n)	1.139	0.740	1.751	0.287
Residents per internal specialists (n)	0.831	0.535	1.290	0.942
Compulsory education only (%)	1.012	0.948	1.080	0.703
Academic degree (%)	0.989	0.943	1.037	0.791
Unemployment rate (%)	0.976	0.754	1.263	0.564
Conventional risk factors				
Hypertension	0.806	0.444	1.464	0.798
Hyperlipidemia	0.587	0.347	0.994	0.016
Diabetes mellitus	1.484	1.021	2.157	< 0.001
Overweight (BMI > 25)	0.706	0.403	1.237	0.072
Smoker	0.962	0.698	1.325	0.005
Family history	0.724	0.336	1.560	0.127
CVD	1.656	0.782	3.510	0.001
PVD	1.794	0.828	3.887	0.003
Age > 62	4.519	2.378	8.586	< 0.001

There was, however, a significant impact of the residential district per se on survival. This can be seen in the Kaplan Meier analysis, plotting the survival of patients per district. The analysis shows a significant difference between the districts (*p*-Value = 0.028). Patients from districts number 13, 14, 18 and 19 had the worst outcome as measured by the duration of survival, whereas patients from districts number 9, 15, 16 and 20 had the best outcome.

## Discussion

This study examines the impact of socio-economic environment and conventional risk factors on the medical outcome for STEMI patients.

The association between conventional risk factors (hypertension, hyperlipidemia, diabetes mellitus, overweight, smoking, family history, CVD, PVD, age) and medical outcomes for STEMI patients have been well documented in several clinical trials [2, 8–10, 12, 13, 16]. Similarly, the importance of the optimal management of conventional risk factors and its benefit for medical outcomes has been confirmed in various studies and can be found in guidelines of the European Society of Cardiology [2, 14].

The socio-economic environment has been shown to be strongly associated with patient risk factors and health outcomes, in general [17, 20, 30] and for CAD [12, 18, 23]. According to our ranking analysis described earlier, districts 1, 8, 9 and 13 form a top group in respect to socio-economic environment, whereas districts 10, 11, 15, 20 and 22 form a bottom group. Of the districts with a high socio-economic environment, the 1st district, corresponding to the “inner city” stands out by leading on all indicators. This main business, shopping and government district is special not the least because it is not really a residential district owing to high rents. Indeed, the high share of patients aged above 62 for the 1st district can be explained by the fact that it is mostly elderly persons benefitting from rent protection who can still afford residence in this area. Nevertheless, doctors tend to do business in the 1st district due to the prestige and closeness to many employers, which explains the low shares of residents per general practitioner and internal specialist. Aside from the inner city, districts number 8, 9, 13 and 19 also feature high incomes, high rates of academic degrees and low rates of unemployment.

The lowest income can be found in the predominantly working class districts number 10, 15 and 20. These districts also have low rates of academic degrees and high rates of compulsory education only. Districts number 15 and 20 also show high rates of unemployment. The 11th district exhibits the highest number of residents per internal specialist and the second highest number of residents per general practitioner. It is also the district with the lowest rate of academic degrees and the highest rate of unemployment. The reason for the poor density of doctors in this district might be the fact, that there is no hospital in the 11th district. A similar phenomenon can be found in the 21st district.

It is notable that across districts there is significant correlation between the socio-economic indicators, including the density of health care provision, which illustrates that socio-economic aspects are tightly interwoven and influence each other.

### Conventional risk factors and socio-economic environment

By considering the joint distribution of SES indicators and conventional risk factors we are able to study at population level the association between SES and risk factors. Generally, the conventional risk factors of this study go along with the literature (see above). All conventional risk factors, aside from hypertension and overweight, were highly significant in relation to the survival of patients, and hypertension shows at least a clear trend with a *p*-value of 0.061 (Table 3).

A clear socio-economic profile of the conventional risk factors is shown in Table 3. Conventional risk factors associated with a good socio-economic profile are hypertension and CVD. The risk factors hyperlipidemia, overweight and current smoking are associated with a poorer SES profile. This suggests that poor socio-economic environments are not uniformly associated with higher conventional risk factors. The above average SES profile associated with the group aged above 62 suggests that on average the elderly (and, thus, those exposed to higher STEMI risks) tend to live in better and potentially protective socio-economic environments. Conversely, patients below 62 tend to be drawn from poorer socio-economic environments. While we cannot claim causality, the association is suggestive of a poor socio-economic environment being conducive to premature (in terms of age) STEMI events.

A particularly interesting finding pertains to the role of diabetes mellitus as a risk factor.

Specifically, patients with IDDM are associated with the highest income and the highest proportion of residents per general practitioner. They also tend to come from districts with a low rate of compulsory education only and a high rate of academic degrees. Conversely, patients with NIDDM tend to come from disadvantaged districts with the lowest income, the highest rate of compulsory education only, the lowest rate of academic degrees and the highest rate of unemployment. NIDDM patients are also associated with the worst proportions of residents per general practitioner and internal specialist, respectively. Notably, IDDM, as compared to NIDDM, is a more serious and advanced stadium of diabetes mellitus. This finding suggests the presence of selection: Patients who benefit from a good socio-economic environment tend to show as STEMI patients only when facing a severe diagnosis, while milder risk factors seem to be under better control. In contrast, patients drawn from poorer environments show as STEMI also under milder conditions, suggesting a weaker control of cardiac risks. Again, we can take this as evidence for a good socio-economic environment having a protective impact.

The study of Jones et al. [18] demonstrated that a low socio-economic status is significantly associated with an adverse prognosis after PCI using mortality as endpoint. It also shows, that patients with a low SES have higher rates of conventional risk factors, which is in line with the correlation between conventional risk factors and socio- economic environment documented in the present study.

### Survival

A COX-regression and a Kaplan Meier survival analysis was conducted to explore the impact of conventional risk factors as well as of the socio-economic environment at a patient’s residential district on individual survival. The results of the COX regression of the conventional risk factors in Table 4 showed that hyperlipidemia, diabetes mellitus, smoking, CVD, PVD and age have a significant influence on survival. Indeed, this goes along with other studies [3, 6–8, 10, 11, 14]. Hypertension, overweight and family history showed no significant influence on survival in the COX regression.

The COX-regression showed no significant impact of the indicators of the socio-economic environment on individual survival (Table 4). Given the expected clear impact of conventional risk factors on survival, which are directly controlled for at the individual level, the finding that the socio-economic environment at the individual’s place of residence has no significant effect on survival would suggest that the socio-economic environment has no direct impact on treatment outcomes beyond determining the patient’s conventional risk. Whether or not this is true is difficult to gauge. On the one hand, there is a positive correlation between socio-economic environment and conventional risk factors at population level; on the other hand, it is well known that this result does not suggest such a relationship at the individual level but may rather be down to compositional effects.

Our analysis revealed, however, a significant variance of survival across the districts (as measured by their postcode) of Vienna (*p*-Value = 0.028). According to the Kaplan Meier analysis the worst survival outcomes are found in districts 13, 14, 18 and 19, whereas the best outcomes are found in districts number 9, 15, 16 and 20. While one might expect that the post code might serve as a summary measure of the socio-economic environment, the apparent lack of correlation between survival outcome and the ranking of districts in terms of their socio-economic environment (13 and 9 among the top-5; 15 and 20 among the bottom 5) is striking. Considering individual districts shows that the 14th district performs worst in terms of the number of residents per general practitioner. In contrast, the 9th district is the second best district in terms of the density of internal specialists. While this may be suggesting a role for primary care provision in terms of explaining survival, the result may also be down to location: The General Hospital of Vienna (AKH Wien) being situated in the 9th district is likely to explain the high density of internal specialists, but it is also indicative of short travelling times. In contrast, the 14th district is located relatively further off. The bad survival rates in districts number 13, 18 and 19 do not go along with the data in this study, as the SES indicators of these districts are above average. Conversely, the unexpectedly good survival rates in districts 15 and 20 are out of line with the SES indicators of these districts being below average. All of this suggests a need for further exploration of possibly unobserved district-specific factors.

### Limitations

This study has a number of limitations. The study is an observational analysis of a single center. The data set includes most clinical variables known. However, the results might be confounded by variables which were not collected. In contrast to the conventional risk factors, it was impossible to collect socio-economic data for each individual patient but only at district level. While we could synchronize the socio-economic environment with the postal code of each patient, the impossibility of directly measuring the SES of a patient rules out the identification of any causal relationship between socio-economic data and survival. This indicates a distinct need for the availability of matched clinical and socio-economic data at individual level.

## Conclusions

This study has investigated the socio-economic environment and the conventional risk factors of patients who underwent PCI after STEMI. The study demonstrated an association between the distribution of socio-economic status and conventional risk factors across districts in Vienna, Austria. The conventional risk factors, in turn, showed significant impact on the survival for STEMI patients.

In summary, the study could provide intuitive evidence on the link between socio-economic environment and conventional risk factors at population level and the link between conventional risk factors and survival both at the population and at the individual level.

The study suggests that knowledge of the socio- economic environment within (residential) districts is important for understanding the prevalence of cardiovascular risk factors among the population at district level. Such knowledge may allow for better prevention strategies and improving the provision of treatment courses for CAD. Therefore SES indicators should be integrated into guidelines, prognostic calculations and management of cardiovascular disease, especially in STEMI patients.

## Abbreviations

ACS: Acute coronary syndrome
AHA: American Heart Association
CABG: Coronary artery bypass grafting
CAD: Coronary artery disease
CCS: Canadian cardiovascular society
CVD: Cerebrovascular disease
ESC: European Society of Cardiology
IDDM: Insulin-dependent diabetes mellitus
MI: Myocardial infarction
NIDDM: Non- insulin-dependent diabetes mellitus
NYHA: New York heart association
PCI: Percutaneous coronary intervention
PVD: Peripheral vascular disease
SCAD: Stable coronary artery disease.
SES: Socio-economic status
STEMI: ST-segment elevation myocardial infarction
UA: Unstable-angina
WHO: World Health Organization

## Authors’ information

CR is a specialist in cardiology at the Medical University of Vienna. RB is a specialist in cardiology at the Hospital of St. John of God in Eisenstadt. MK is a research group leader at the Wittgenstein Centre (IIASA, OEAW/VID, WU) and Vienna Institute of Demography.
